# Supplementary material for: Three-Questions-Method for Coping with the Emotional Burden of Nurses and Nursing Students during COVID-19
Source: Int J Environ Res Public Health. 2022 May 27;19(11):6538. doi: 10.3390/ijerph19116538 (PMC9180629; doi:10.3390/ijerph19116538)
Supplement: Supplementary file 1 [file ijerph-19-06538-s001.zip › ijerph-1711756-supplementary.pdf]

Supplementary Materials

Figure S1. Study phases, objectives, and participants.

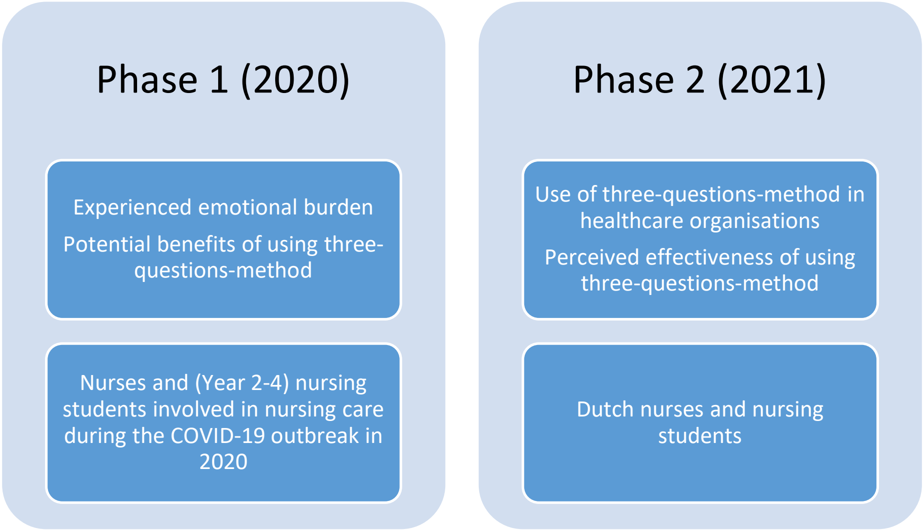

Figure S2. Phase 1 interview guide focus group.

1. How did you experience working during the COVID-19 pandemic?
2. How did you feel while working during the COVID-19 pandemic?
3. Introduction of three-questions-method: Looking back at your experiences during the COVID-19 outbreak. How would using the method help you cope with the emotional burden?
4. Would it offer you an added value ?
   -During a shift/transfer?
   -During education/intervision?
5. How important is peer support in coping with your experiences during the COVID-19 outbreak?
6. Are there any topics that we have not covered in the conversation that you think are important to mention?

Figure S3. Phase 2 interview guide semi-structured interviews.

Users three-questions-method

1. How did you come across the three-questions-method? Or how did you first hear about the three-questions-method?
2. How did you experience the three-questions-method?
3. How frequently (daily, weekly, monthly) did you and your colleagues use the three-questions-method in your department?
4. At what times did you and your colleagues use the three-questions-method?
5. Is this used individually or in groups?
6. Is it used organization wide and in what frequency?
7. What are facilitating and hindering factors in using the three-questions-method?

8. How do you feel the three-questions-method contributes to your emotional well-being?
9. Why would you recommend the three-questions-method? Why not?
10. Are there any topics that we have not covered in the conversation that you think are important to mention?

Non-users three-questions-method

1. How did you experience working during the COVID-19 pandemic?
2. How did you feel while working during the COVID-19 pandemic?
3. Introduction three-questions-method: Looking back at your experiences during the COVID-19 outbreak. How would using the method help you cope with the emotional burden?
4. Would it offer you an added value ?
5. What could be facilitating and hindering factors for using the three-questions-method?
6. How would you feel if colleagues asked you these questions?
7. How frequently would you use it?
8. How often would you like to be asked these questions?
9. Why would you recommend the three-questions-method? Why not?
10. Are there any topics that we have not covered in the conversation that you think are important to mention?
